# Novel contouring method for optimizing MRI flow quantification in patients with aortic valve disease

**DOI:** 10.1007/s10554-023-03036-0

**Published:** 2024-01-30

**Authors:** Malek M. Yaman, Michael Chetrit, Jennifer Bullen, Michael A. Bolen, Scott D. Flamm, Deborah Kwon

**Affiliations:** Cleveland Clinic Foundation, 9500 Euclid Ave., Cleveland, OH 44195 USA

**Keywords:** MRI, Blood flow, Aorta, Aortic valve, Mitral regurgitation

## Abstract

Optimizing MRI aortic flow quantification is crucial for accurate assessment of valvular disease severity. In this study, we sought to evaluate the accuracy of a novel method of contouring systolic aortic forward flow in comparison to standard contouring methods at various aortic levels. The study included a cohort of patients with native aortic valve (AoV) disease and a small control group referred to cardiac MRI over a 1-year period. Inclusion criteria included aortic flow quantification at aortic valve and one additional level, and no or trace mitral regurgitation (MR) documented both by the MRI AND an echocardiogram done within a year. In addition to flow quantification with standard contouring (SC), a novel Selective Systolic Contouring (SSC) method was performed at aortic valve level, contouring the area demarcated by the AoV leaflets in systole. The bias in each technique’s estimate of aortic forward flow was calculated as the mean difference between aortic forward flow and left ventricular stroke volume (LV SV). 98 patients (mean age 56, 71% male) were included: 33 with tricuspid and 65 with congenitally abnormal (bicuspid or unicuspid) AoV. All methods tended to underestimate aortic forward flow, but the bias was smallest with the SSC method (p < 0.001). Therefore, SSC yielded the lowest estimates of mitral regurgitant volume (4.8 ml) and regurgitant fraction (3.9%) (p < 0.05). SSC at AoV level better approximates LV SV in our cohort, and may provide more accurate quantitative assessment of both aortic and mitral valve function.

## Introduction

Cardiac MRI (CMR) provides comprehensive assessment of aortic valve (AoV) disease and is increasingly utilized [1]. It provides excellent assessment of AoV morphology and function, evaluation of co-existing aortopathy, and gold standard quantification of left ventricular size, ejection fraction, and mass index, which are important for clinical decision making and timing of AoV interventions [2].

Most commonly, aortic flow quantification is performed at the level of sinotubular junction (STJ) and/or mid ascending aorta (AAo), although other locations (distal AAo, AoV, and LVOT) have been utilized [3–5]. In clinical practice, flow quantification performed at various levels can yield discrepant values [3, 4, 6], and there is still debate regarding which level most accurately reflects aortic flow.

Optimizing aortic flow quantification is crucial, since it is used in clinical practice for both assessment of AoV function and indirect calculation of mitral regurgitant volume. This becomes more paramount in patients with concomitant AoV and mitral valve (MV) disease, with higher potential for errors due to altered flow patterns in the ascending aorta, as demonstrated by 4D CMR studies [7].

**Fig. 1:**
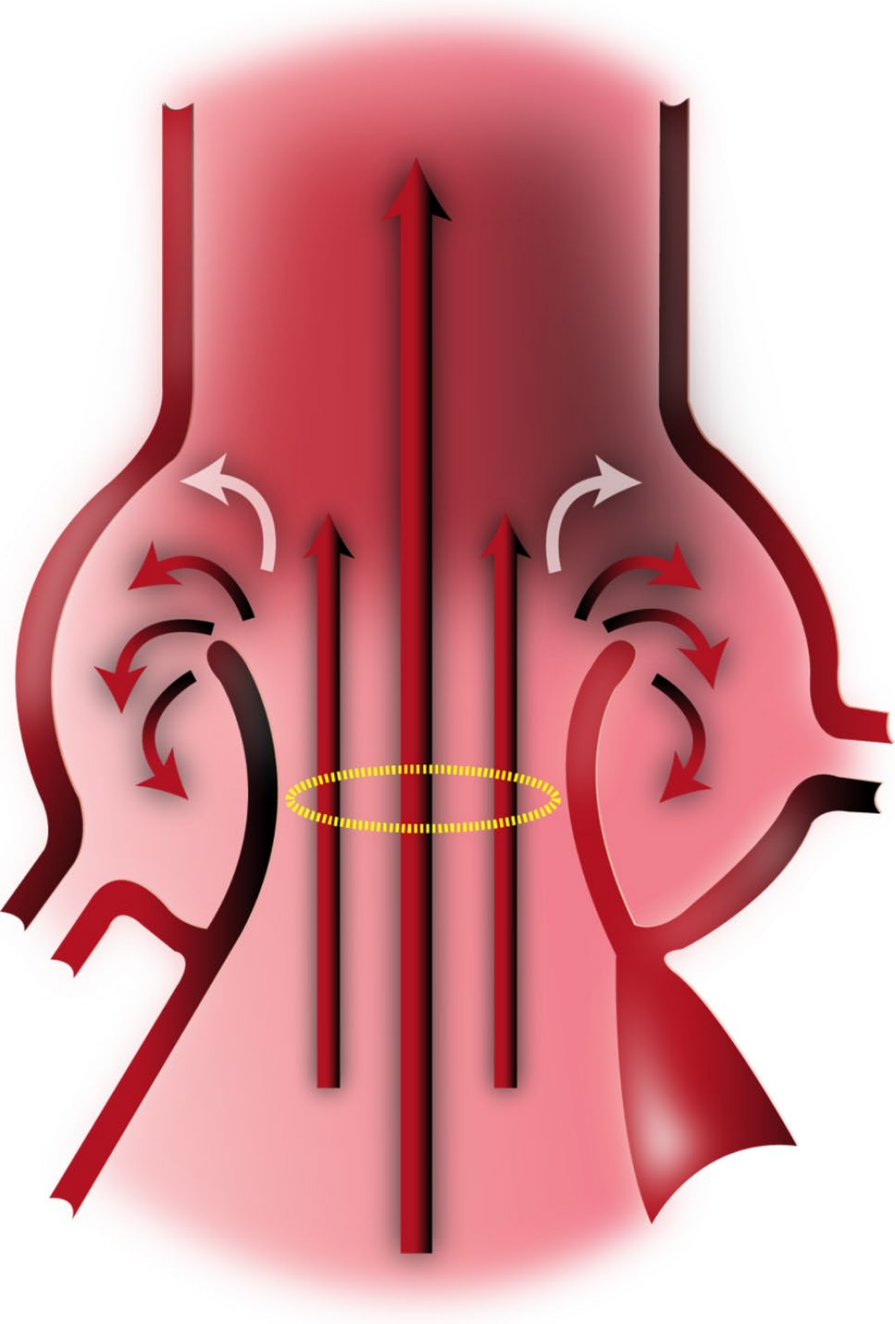
Schematic showing antegrade jet going through AoV orifice, surrounded by secondary flow jets

Our group has incorporated flow quantification at AoV level to clinical CMR protocols, with favorable initial results. In this study, we wished to further explore the comparison of forward flow at various levels in patients with congenital or degenerative AoV disease. Our primary goal was to assess the accuracy of a *novel method of systolic flow contouring* at the level of the AoV, consisting of *selective systolic contouring* (SSC) of the area *within* the confines of the AoV leaflets (Fig. 1). We hypothesized that SSC method would yield more accurate forward flow quantification than standard contouring (SC). Secondary goals included comparing aortic regurgitant volume and fraction from various methods, and the impact of different methods on quantification of mitral regurgitation.

## Materials and methods

An IRB-approved, retrospective study with waiver of informed consent analyzed imaging from all consecutive patients over a 12 month period who had a CMR for native AoV disease, and met the following criteria. (1) Aortic flow quantification obtained at AoV level plus at least 1 other level (STJ and/or AAo). (2) Complete LV volumetric assessment (short axis cine stack). (3) No ventricular septal defect (VSD), and no or trace MR documented both by the MRI and an echocardiogram done within a year of the MRI (with no interceding cardiac interventions) to ensure excellent expected correlation between LV SV and aortic forward flow. (4) No subaortic stenosis. (5) No supravalvar aortic stenosis or significant coarctation (clinical pressure gradient > 20 mm Hg). (6) No prior aortic valve interventions. (7) High quality complete MRI dataset.

**Fig. 2:**
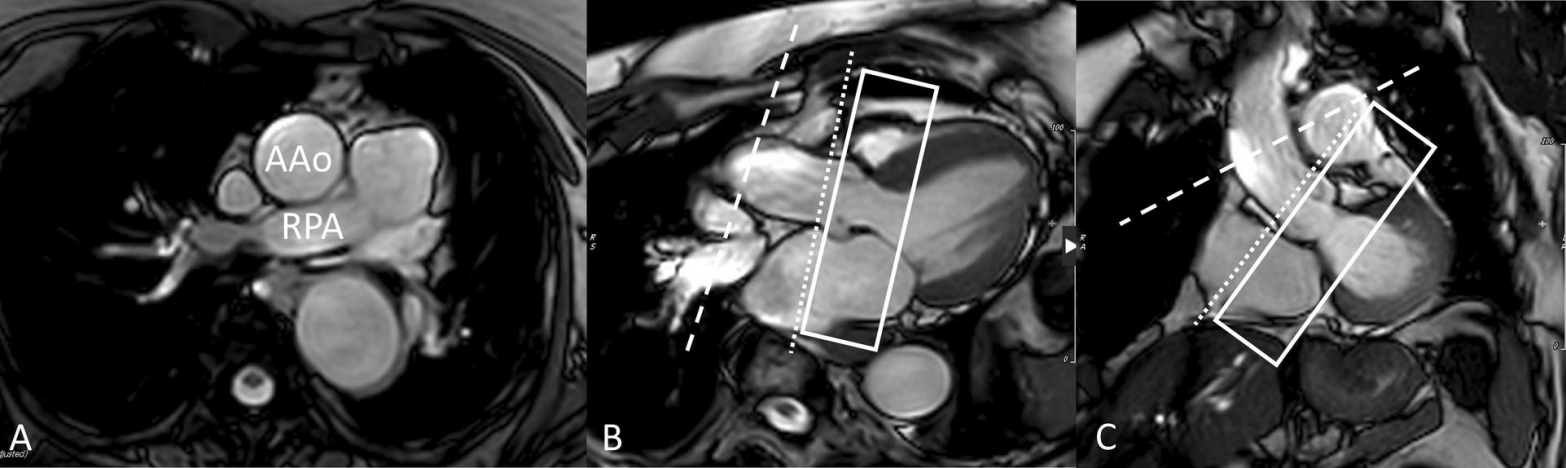
**A** shows axial slice of AAo at the level of the right pulmonary artery (RPA). **B** (LV 3 chamber view) and **C** (LVOT view) show the slice position of phase contrast velocity encoded sequences at the level of the AAo (dashed line) and STJ (dotted line). The AoV short axis stack (rectangle) consisted of a 5–8 slice SSFP cine stack with a plane parallel to the AoV hinge points as seen on 3 chamber and LVOT views

Patients underwent CMR on a commercially available 1.5 Tesla scanner (Philips Achieva, 200 mT/m/ms gradient, 8-channel SENSE-XL-Torso coil, Philips Medical Systems, Best, The Netherlands) or 3 Tesla scanner (Philips Ingenia, 200 mT/m/ms gradient, 16-channel SENSE-XL-Torso coil, Philips Medical Systems, Best, The Netherlands). After the scout images, an axial, single shot, steady-state free precession (SSFP), non-electrocardiogram-gated stack of images was performed through the thorax during an end-expiratory breath-hold. Standard electrocardiogram-gated 2-, 3-, 4-chamber and short-axis as well as LVOT SSFP cine images were obtained using the following parameters: repetition time 2.5 ms, TE 1.3 ms, flip angle 70°, field of view 300_260 mm, matrix 128_128, SENSE reduction factor 1.3, 30 phases per cardiac cycle, and a typical breath-hold of approximately 12 s on the 1.5 T scanner, and repetition time 3 ms, TE 1.5 ms, flip angle 55°, field of view 320_320 mm, matrix 184_180, SENSE reduction factor 2, 30 phases per cardiac cycle, and a typical breath-hold of approximately 10 s on 3T scanner. In addition, a 5–8 slice SSFP cine stack (which we refer to as AoV Short Axis Stack) was obtained across LVOT, AoV, and aortic root, with a plane parallel to the AoV hinge points based on two double oblique LVOT cine images (Fig. 2). This stack was used for two purposes: (1) define AoV morphology, and (2) choose the slice position that best visualizes the aortic leaflets throughout systole, which was then used to perform flow quantification at the AoV.

Through-plane electrocardiographically gated Phase Contrast Velocity Encoded sequences were done during a breath-hold at end expiration at AoV level, plus one or both of the following two locations: STJ, and/or mid AAo. Flow at AoV level was planned by choosing the slice position from the above-described AoV Short Axis Stack which best shows the entirety of the aortic valve leaflets throughout all of systole. If two slices met this criterion, the technologist chose the slice that showed the leaflet boundaries most clearly. Flow at the STJ was systematically planned by placing the slice at the sinotubular junction such that it is simultaneously perpendicular to the aorta as seen on two orthogonal planes (3 chamber view and LVOT view, as shown in Fig. 2). AAo flow was planned by initially selecting an axial slice of the AAo at the level of the right pulmonary artery (RPA), then adjusting the slice orientation to also be simultaneously perpendicular to the aorta on the above-mentioned orthogonal views (Fig. 2). Typical sequence parameters were repetition time 4.8 ms, echo time 2.8 ms, flip angle 12°, field of view 300_260 mm, matrix 128_98, SENSE reduction factor 2, 30 phases per cardiac cycle, and a typical breath-hold of 12 s. An encoding velocity of 200 cm/s was initially used, which was increased by 100–200 cm/s increments in case of aliasing.

**Fig. 3:**
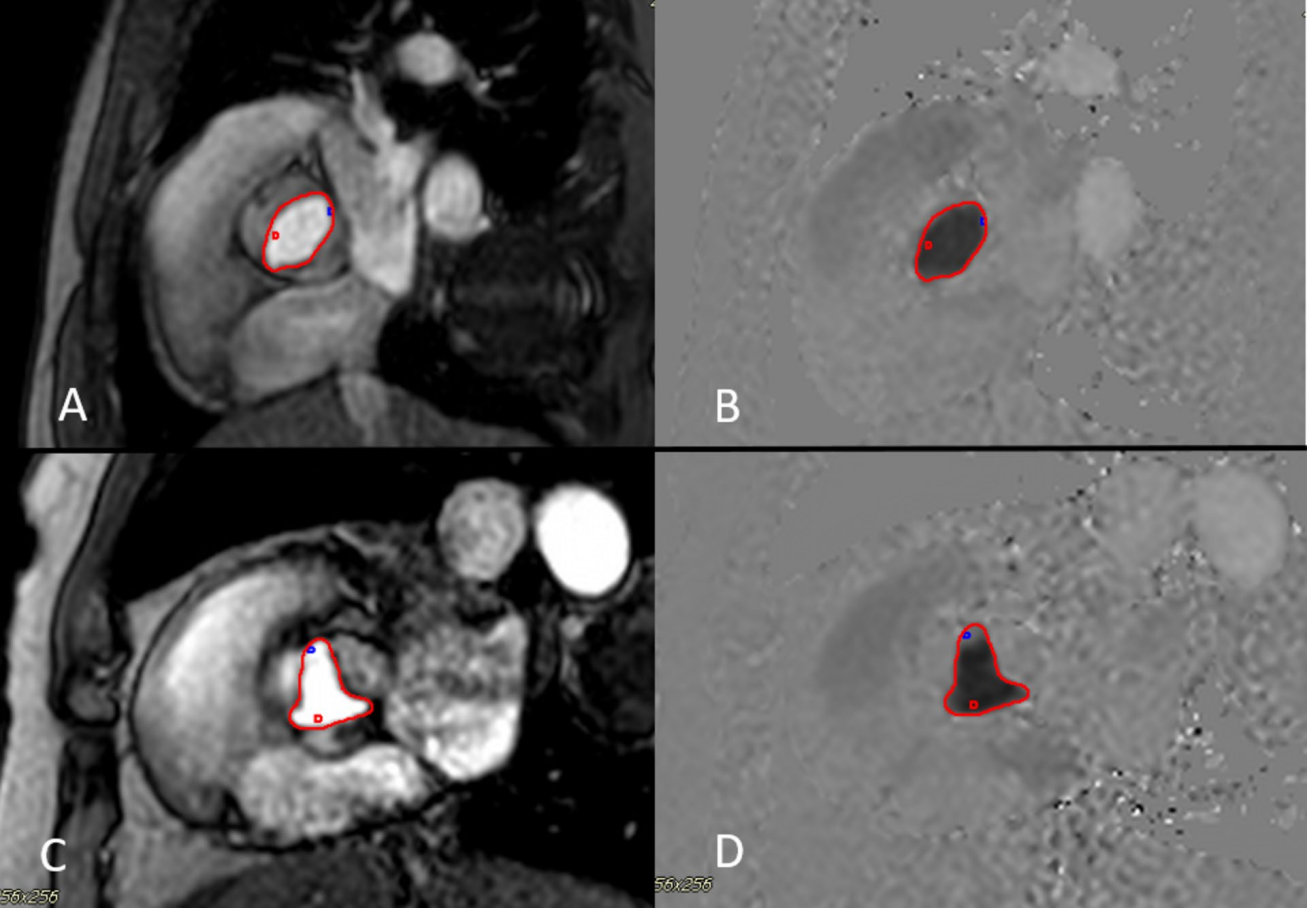
SSC at AoV level demonstrated in patient with bicuspid AoV (**A** magnitude image, **B** phase velocity map image), and in another patient with a tricuspid AoV (**C** magnitude image, **D** velocity image). Note that the proper choice of aliasing velocity (just above Vmax) creates more visual contrast on the velocity map images that aids in contour placement

**Fig. 4:**
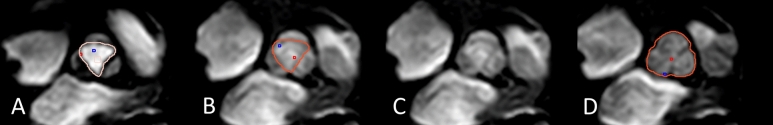
SSC contouring at AoV level is demonstrated on magnitude images in a patient with tricuspid AoV, during select representative phases of the cardiac cycle: **A** (peak systole), **B** (end-systole), **C** (end-systole, contour omitted to show how the leaflet borders were visualized for demarcation), and **D** (mid-diastole, demonstrating how the whole lumen, not selective contouring, was done during diastole)

All studies were analyzed by one investigator (MY). Post-processing was performed using a dedicated software (CVI42 v. 5.1.0, Circle Cardiovascular Imaging Inc., Calgary, Canada). LV volumes included the LVOT, trabeculated myocardium and papillary muscles. Flow sequences at various levels were contoured to yield forward flow, reverse flow, and regurgitant fraction, using standard contouring (SC) methods, by placing contours around the entire cross section of the aortic lumen, as previously described in the literature [2, 3, 5]. In addition to SC at all levels, a novel contouring method was performed at AoV level, consisting of SSC of the area demarcated by the AoV leaflets, aided by both the magnitude and phase velocity map images from Phase Contrast acquisition. During diastole, the entire aortic cross section was included in the flow, similar to SC. Figure 3 shows examples of SSC for a bicuspid AoV (A and B) and tricuspid AoV (C and D). Care should be taken to accurately delineate the aortic valve leaflets during systole, as the leaflets move and change configuration, especially in early systole and end-systole as the valve is opening or closing with low velocity flow, whereby the magnitude images become particularly helpful to demarcate the valve opening. Figure 4 shows an example of how SSC was done on two representative phases in systole (peak systole, and end-systole right before valve closes, as well as during mid-diastole), in a patient with tricuspid AoV.

Additional MRI data collected included AoV morphology, AoV area by planimetry as previously described [1], and maximum aortic root and ascending aorta diameters (measured on multiplane reconstruction of 3D SSFP sequences).

As a quality measure, 10% of the studies were randomly selected and blindly contoured by another investigator, DK, to assess the agreement between two readers with respect to forward flow and regurgitant fraction by SSC.

To assess the feasibility of SSC in patients without AoV disease, 10 controls were included who had a CMR between June and July 2020, and had: MRI-derived LVEF ≥ 55%, trileaflet AoV without stenosis or regurgitation, no VSD and no or trace mitral regurgitation verified by echocardiography and CMR, and Phase Contrast Velocity Encoded sequence performed at AoV plus one other level. Contouring and analysis was done using same methods described above.

Echocardiographic data which was collected included AoV morphology, maximum velocity (Vmax), and degree of aortic and mitral regurgitation assessed per American Society of Echocardiography.

### Statistical analysis

The primary aim of this analysis was to assess the accuracy of four CMR aortic flow measurement techniques. Since included patients had little to no MR, true aortic forward flow was assumed to be equal to LV SV. The bias in each technique’s estimate of aortic forward flow ($\overline{x }$) was therefore calculated as the mean difference between aortic forward flow and stroke volume. Limits of agreement (LOAs) [8], which characterize the precision of the technique as well as its bias, were also calculated. The LOAs were defined as $\overline{x }\pm 1.96s$, where $s$ is the standard deviation of the differences between aortic forward flow and stroke volume.

Several variables were assessed as potential predictors of increased discrepancy between LV SV and aortic forward flow: Vmax by Echo, valve morphology, CMR-derived LVEF, aortic root max dimension, AAo max dimension, and AoV planimetry. A series of simple linear regression models were fit, where the discrepancy between LV SV and aortic forward flow was the dependent variable. Each MRI measurement technique was assessed separately, and Holm’s adjustment method was used to maintain a family-wise error rate of 0.05.

To assess the agreement between the two readers with respect to SSC in 10 randomly selected individuals, the intra-class correlation coefficient was computed. A two-way random effects model was used, assuming a single rater.

## Results

98 patients (mean age 49 years, 71% male) were included: 33 with tricuspid AoV, and 65 with congenitally abnormal AoV (59 bicuspid, 6 unicuspid AoV). Demographics and patient characteristics are summarized in Table 1.

**Table 1:** Summary of patient characteristics among those with aortic valve disease

	Patients with aortic valve disease (N = 98)
Age (years) at MRI [mean (SD)]	48.9 (18.6)
Male (%)	71 (72.4)
Aortic valve morphology (%)	
Unicuspid	6 (6.1)
Bicuspid	59 (60.2)
Tricuspid	33 (33.7)
Grade of aortic regurgitation by echo (%)	
< Moderate	43 (43.9)
Moderate	43 (43.9)
Severe	12 (12.2)
Vmax (m/s) by echo* [mean (SD)]	2.2 (0.9)
Severity of aortic stenosis by echo (%)	
Vmax < 3 m/s	81 (82.7)
Vmax 3–4 m/s	12 (12.2)
Vmax > 4 m/s	5 (5.1)
BSA (m^2^) [mean (SD)]	2.0 (0.3)
LVEF (%) by MRI [mean (SD)]	60.1 (7.3)

**Fig. 5:**
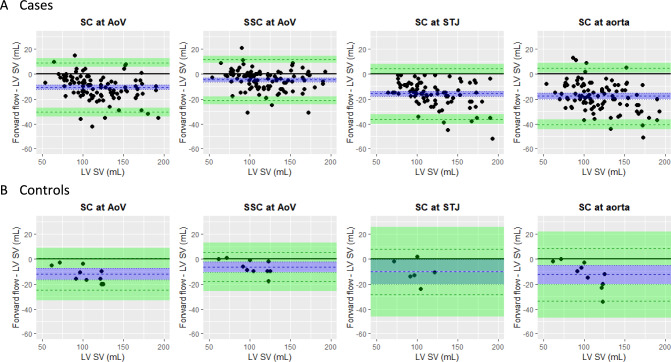
Bland Altman plots. Points above solid black like indicate patients with forward flow > LV SV, while points below the line indicate patients with forward flow < LV SV. Blue dashed line = mean difference between forward flow and LV SV across patients (i.e. bias of flow measurement). Blue shaded area = 95% CI for bias. Green dashed lines = limits of agreement between forward flow and LV SV. Green shaded areas = 95% CI for limits of agreement

**Fig. 6:**
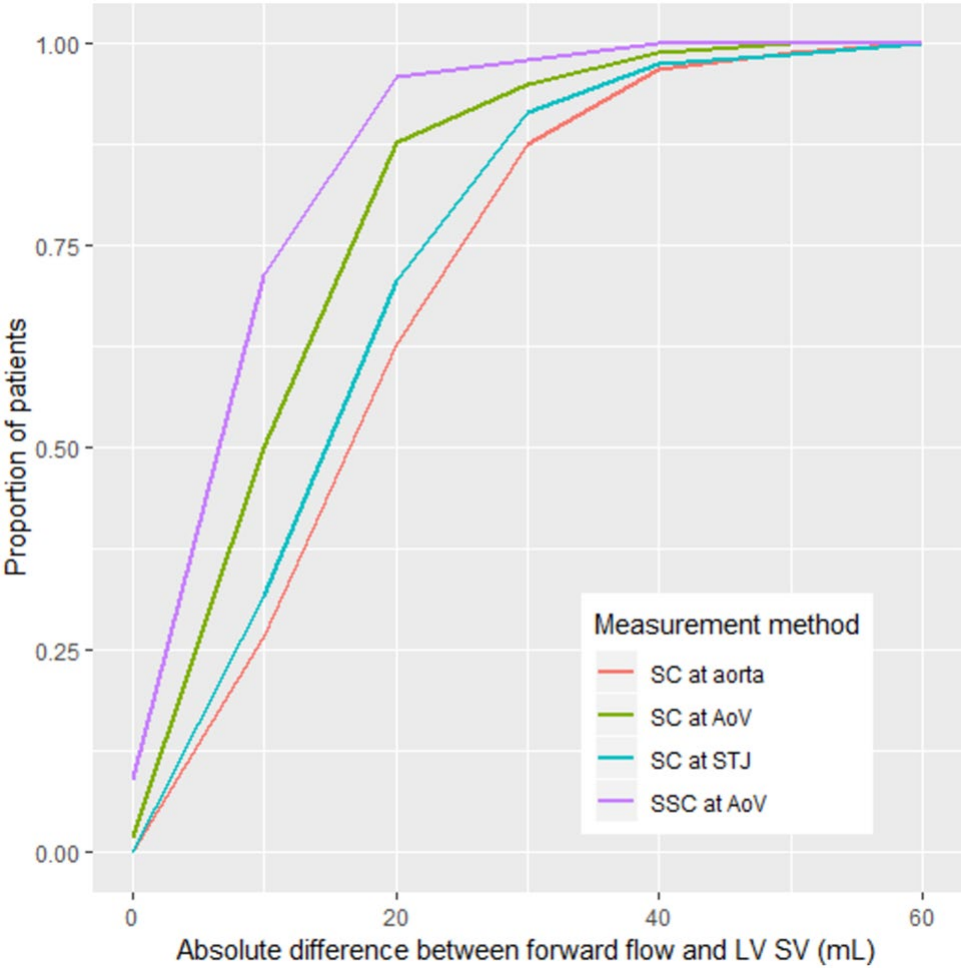
Proportion of cases where the absolute difference between forward flow and LV SV was less than or equal to a given threshold. N = 98 patients for SC at AoV and SSC at AoV, 82 patients for SC at STJ, and 97 patients for SC at aorta

Bland Altman plots for SC at various levels and SSC at AoV level are presented in Figure 5. All methods tended to underestimate aortic forward flow (Table 2); the bias was smallest with SSC (p < 0.001). On average, the SSC method underestimated the aortic forward volume by 5 ml (95% CI 3,7). The absolute difference between SSC forward flow and stroke volume was 18 ml or less for 95% of patients. In comparison, the absolute difference between forward flow by SC and stroke volume for 95% of patients was 29 ml or less at AoV, 35 ml or less at STJ, and 36 ml or less at AAo level (Fig. 6).

**Table 2:** Bias and limits of agreement (LOAs) for four aortic flow measurement techniques

Aortic flow measurement technique	N	Bias (ml)		Lower LOA (ml)		Upper LOA (ml)	
		Estimate	95% CI	Estimate	95% CI	Estimate	95% CI
(A) Cases							
SC at AoV	98	− 11	(− 13, − 9)	− 31	(− 34, − 27)	9	(5, 12)
SSC at AoV	98	− 5	(− 7, − 3)	− 21	(− 24, − 18)	12	(9, 14)
SC at STJ	82	− 16	(− 18, − 14)	− 36	(− 40, − 32)	4	(0, 8)
SC at aorta	97	− 18	(− 20, − 16)	− 41	(− 45, − 37)	5	(1, 9
(B) Controls							
SC at AoV	10	− 12	(− 17, − 8)	− 25	(− 33, − 17)	1	(− 8, 9)
SSC at AoV	10	− 6	(− 11, − 2)	− 18	(− 26, − 11)	5	(− 2, 13)
SC at STJ	6	− 10	(− 20, − 1)	− 28	(− 46, − 11)	8	(− 10, 26)
SC at aorta	10	− 13	(− 20, − 5)	− 34	(− 47, − 20)	8	(− 5, 22)

**Fig. 7:**
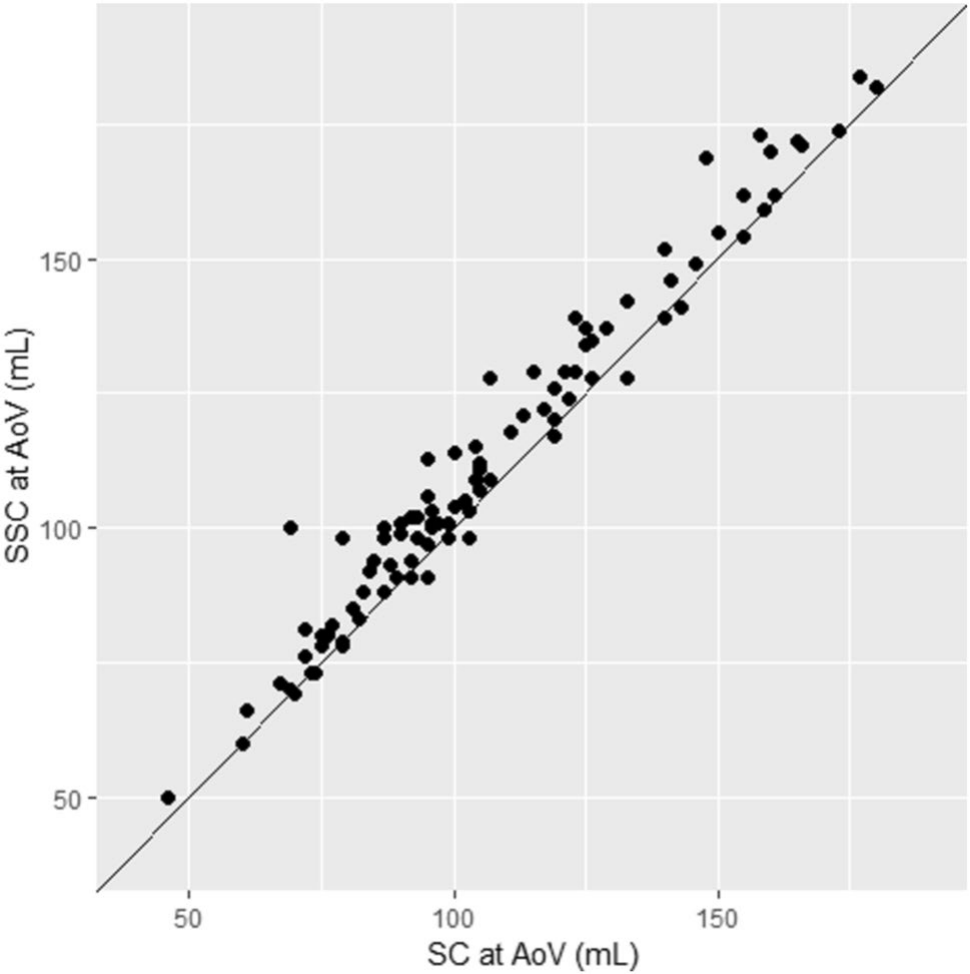
Forward flow derived by SSC versus SC at AoV level. SSC gave an equal or larger value than the SC method for 86 of the 98 patients (88%)

Figure 7 shows that SSC results in equal or larger forward flow estimate than that derived by SC at AoV level in 88% of cases, implying zero or net negative systolic flow *outside* the valve boundaries in those cases. In the remaining 12 cases in whom SSC yielded lower forward flow than SC, the discrepancy was 2 ml or less in 9/12 cases, and 4–5 ml in the remaining three. The latter cases were reviewed and the underestimation of flow by SSC contouring appeared to be related to a combination of factors including a very irregular shaped valve border, vague/poorly demarcated valve border of at least one portion of the valve, and/or slice sub-optimally placed just above leaflet tips during a portion of systole.

A number of factors that could potentially predict a wider discrepancy between stroke volume and aortic forward flow for the four MRI measurement techniques were assessed. These potential factors were echo-derived Vmax, AoV morphology, LVEF, aortic root and AAo maximum diameters, and AoV planimetry. After adjustment for multiple comparisons, the only statistically significant associations were between increasing aortic root maximum diameter and larger bias via SC at the valve (r = − 0.29, 95% CI − 0.46, − 0.09, p = 0.035) and STJ (r = − 0.33, 95% CI − 0.52, − 0.11, p = 0.023).

The impact of contouring method on quantification of aortic regurgitation is summarized in Table 3, which shows that there was a statistically significant difference in aortic regurgitant volume (RV) across the three measurement levels (p < 0.001). During follow up pairwise comparisons, SC at AAo was significantly lower than both SC/SCC at AoV (< 0.001) and SC at STJ (p < 0.001). The difference between SC at STJ and SC/SSC at AoV was not statistically significant (p = 0.124). On the other hand, there was no statistically significant difference in aortic regurgitant fraction (RF) across the four measurement methods (p = 0.315).

**Table 3:** Aortic regurgitant volume (RV) and regurgitant fraction (RF) using the four measurement methods

	SC at AoV	SSC at AoV	SC at STJ	SC at AAo	p
# Patients	98	98	82	97	
Aortic RV [mean (SD)], ml	20.8 (21.8)	20.8 (21.8)	19.8 (20.2)	15.1 (16.7)	< 0.001
Aortic RF [mean (SD)], %	18.7 (16.7)	16.5 (13.7)	17.0 (13.8)	19.5 (24.2)	0.315

Table 4 shows how the differences between the aortic forward flow among the various methods impacts estimates of mitral RV and RF. There was a statistically significant difference in mean mitral RV across the four measurement methods (p < 0.001); all follow up pairwise comparisons had adjusted p-values < 0.05, with SSC yielding the lowest estimate of mean mitral RV (4.8 ml), compared to 10.8 ml, 16.1 ml, and 17.9 ml using SC at AoV, STJ, and AAo, respectively. Given that all patients were selected with no more than trace mitral regurgitation, these findings suggest that all other methods besides SSC tend to overestimate the degree of mitral regurgitation. Similar findings were encountered when mitral RF and corrected RF were compared between the various methods (corrected mitral RF takes into account aortic regurgitant volume so that the denominator better approximates mitral inflow volume), showing again lowest mitral RF and corrected RF for SSC method.

**Table 4:** Mean mitral regurgitant volume (RV), regurgitant fraction (RF) and corrected RF, as derived from the four different methods

	SC at AoV	SSC at AoV	SC at STJ	SC at AAo	p
# Patients	98	98	82	97	
Mitral RV [mean (SD)], ml	10.8 (10.1)	4.8 (8.4)	16.1 (10.3)	17.9 (11.6)	< 0.001
Mitral RF [mean (SD)], %	8.9 (8.5)	3.9 (7.6)	13.5 (7.4)	15.3 (9.7)	< 0.001
Corrected mitral RF [mean (SD)]	10.8 (10.0)	4.7 (8.7)	15.9 (9.2)	17.4 (11.0)	< 0.001

Mitral RV was calculated using the formula LV SV − aortic forward flow. Mitral RF was calculated using the formula $$\frac{\text{LV \,SV}-\text{aortic\, forward\, flow}}{\text{LV \,SV}}\times 100\%$$. Corrected mitral RF was calculated using the formula $$\frac{\text{LV\, SV}-\text{aortic\, forward\, flow}}{\text{LV \,SV}-\text{aortic\, regurgitant\, volume}}\times
100\text{\%}$$. All follow up pairwise comparisons had adjusted p-values < 0.05, *except* corrected mitral RF derived from SC at STJ vs SC at AAo

Finally, to assess the reproducibility of performing the SSC method, the intra-class correlation coefficient was computed for the 10% randomly selected studies which were blindly contoured by another investigator, DK, with respect to forward flow SSC and regurgitant fraction. There was excellent absolute agreement between the two readers (MY and DK) with respect to forward flow SSC (ICC = 0.92, 95% CI 0.72, 0.98, p < 0.001) and regurgitant fraction (ICC = 0.97, 95% CI 0.74, 0.99, p < 0.001).

### Controls

Table 2B and Fig. 5B demonstrate, in 10 control patients, the difference between aortic forward flow and LV SV using all four contouring methods. Again, all four methods tended to underestimate aortic forward flow, but the bias was smallest with the SSC (6 ml). The LOAs indicate that the SSC method is expected to underestimate aortic flow by no more than 18 ml and overestimate aortic flow by no more than 5 ml for roughly 95% of patients. Findings from this small control group suggests that SSC is probably applicable for patients without aortic valve disease, though the small sample size of this control precludes further statistical analysis.

## Discussion

**Fig. 8:**
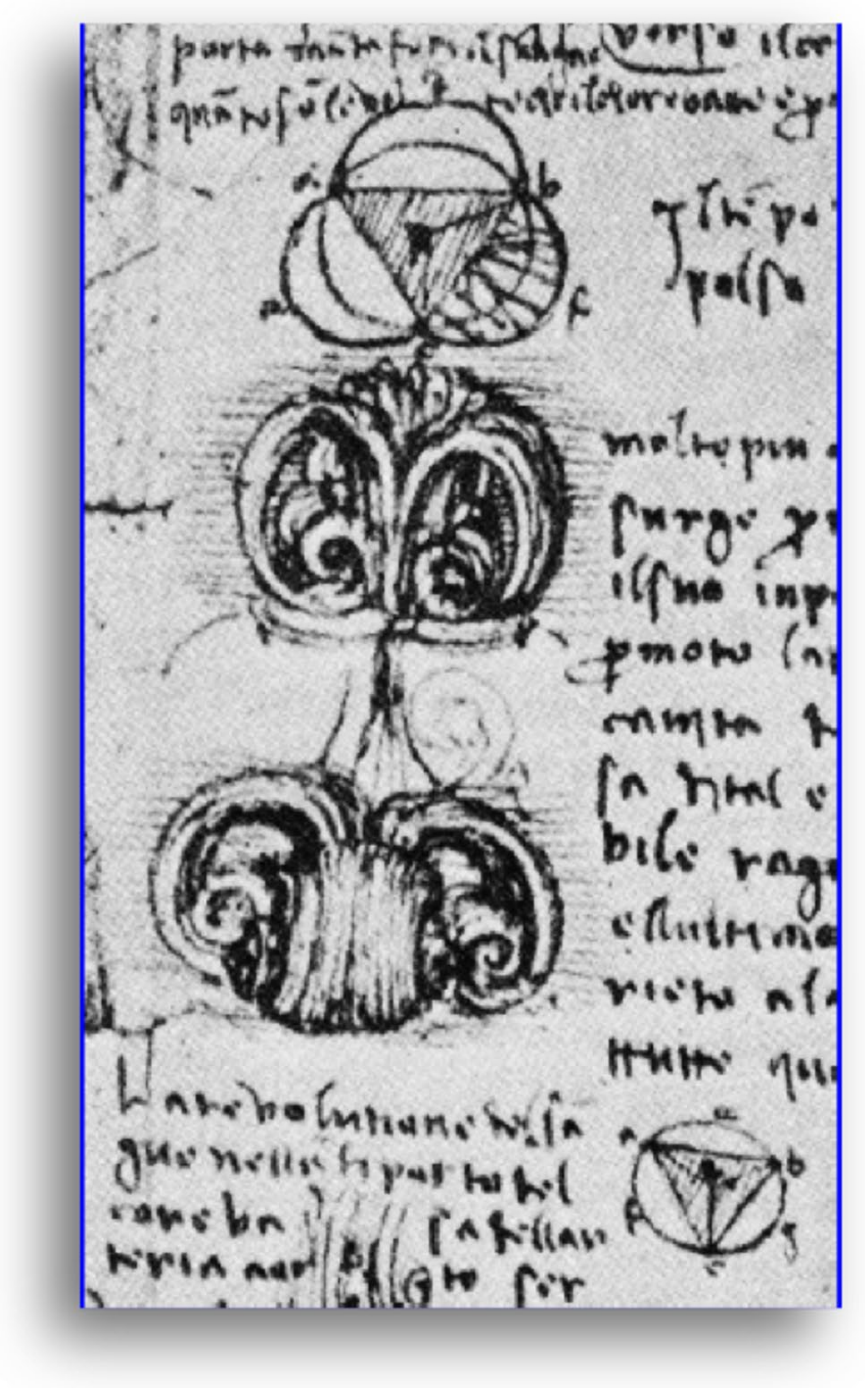
Excerpt from Leonardo Da Vinci’s notebook, showing aortic flow vortices closing the aortic valve from the side (image used with permission of the Royal Collection Trust/ © Her Majesty Queen Elizabeth II 2021)

In this investigation, we found that a novel SSC contouring at AoV level correlated best with LV SV in patients with no more than trivial mitral regurgitation. We believe SSC at AoV level makes physiologic sense, since blood ejected by LV should flow between the leaflets. SSC excludes any swirling flow eddies in the aortic sinuses during systole (Fig. 1), which may later serve in early diastole to efficiently close aortic leaflets, as first described by Leonardo DaVinci [9] (Fig. 8), and suggested in more recent research [10–12]. Figure 7 indicates there may be *net negative flow* in the extravalvular area of the aortic root in 88% of our patients. In the remaining 12%, SSC yielded lower forward flow than SC, though the discrepancy was small, either representing variant flow in the root, suboptimal slice placement, or inherent difficulty delineating the valve contour in some patients with markedly abnormal valves.

The study by Bertelsen et al. [4], which only included patients > 70 years old and excluded patients with aortic stenosis, found that forward flow at ST junction was 13–16% lower than at valve level, more than could be accounted for by coronary artery flow (estimated around 5% [13]). In comparison, forward flow at valve level was closest to LV SV, but still significantly lower (by 8%). Those findings were very similar to the degree of underestimation in our study with SC at AoV and STJ. Muzarelli et al. [3] compared forward flow at various levels (LVOT, AoV, and AAo) with LV SV in patients with bicuspid aortic valves and controls. They found that AAo forward flow underestimated LV SV mildly (by median of 9%) in controls and more significantly (by median of 22%) in those with bicuspid valves. This study was smaller than the current study (22 bicuspid, and 20 controls). Only 2 patients had severe AS; the remaining patients had *no* aortic stenosis. Our study shows the applicability of our findings to patients across a wider spectrum of aortic stenosis (with 17% having moderate to severe stenosis) and regurgitation (with 56% having moderate to severe regurgitation). Unlike the above two studies, our study relied not only on MRI but also on echocardiography to exclude any patients with more than trivial mitral regurgitation, as echocardiography may be more sensitive in detecting milder MR jets than MRI cine images. We opted to include patients with trace MR as very few patients in clinical practice have complete absence of any MR. The presence of trace MR would be expected to cause a discrepancy between LV SV and aortic forward flow that us no more than a few milliliters. Finally, our study, to our knowledge, is the first to use SSC.

Our study showed that SC at STJ and AAo underestimated LV SV by 16% and 15%, respectively, in tricuspid aortic morphology, and 16% and 20%, respectively, in unicuspid/bicuspid aortic valve morphology. This underestimation is likely due to multiple factors, but we suspect that eccentric and complex flow patterns play a major role, with antegrade flows that may not be parallel to the lumen of the vessels and secondary currents which may have a negative direction. Other factors include intravoxel dephasing, or being distal to coronary artery origins. Contijoch et al. [14] showed, using 4D flow, that vortical flow can account for the flow inconsistencies in the AAo; higher variability of flow measurements in the AAo was seen in patients with moderate or severe vorticity, particularly when the vorticity extends more distally. The authors suggested that vorticity visualization may be used to help guide optimal location for flow quantification.

Given that SSC had the highest correlation with LV SV, it also yielded the lowest estimates of mitral RV and RF compared to SC methods. Since all patients were selected with no more than trace mitral regurgitation, all SC methods likely overestimated the degree of mitral regurgitation. This suggests that SSC method may be a robust method for estimating the degree of mitral regurgitation in patients with concomitant aortic and mitral valve disease.

The current study showed no statistically significant difference in aortic RF derived from the various methods, likely because the contouring method impacted both the numerator (aortic RV) and denominator (aortic forward volume). Therefore, SSC appears to provide an advantage in better estimating the degree of aortic forward flow and, therefore, mitral regurgitation, without significantly impacting the estimate of aortic RF compared to other methods.

### Study limitations

The study was carried out at a single tertiary care center, with all scans performed in a Cardiovascular Imaging Lab using two dedicated CMR scanners. Further studies will be needed to verify the reproducibility of these findings within various types of practices with different referral patterns and range of aortic valve pathology, as well as applicability to different scanners. The study is also limited by its retrospective nature, though all contours were prospectively performed. Another limitation is that we did not correct for potential phase offset errors. We relied on previous work from our center [15] which showed that 80% of patients assessed using water phantoms had less than 0.6 cm/s offset velocity in the ascending aorta. It is likely that any underlying phase offset errors affected the various methods for flow quantification similarly. Other limitations include the potential effect of inclusion of papillary muscles and trabeculations within the LV cavity on LV volumes, as well as the use of relatively high encoding velocity of 200 cm/s as a starting velocity, with 100 cm/s increments, which may underestimate low velocity flows.

## Conclusion

In conclusion, a novel selective systolic contouring method is practical and reproducible, and appears to yield a more accurate assessment of aortic forward flow than traditional methods in patients with a wide range of aortic valve pathology. This can help mitigate potential errors in estimating the degree of mitral regurgitation. More studies are needed to verify the reliability and reproducibility of this method, and further assess it in patients with concomitant mitral valve disease.
